# Characterization of Face-Selective Patches in Orbitofrontal Cortex

**DOI:** 10.3389/fnhum.2016.00279

**Published:** 2016-06-14

**Authors:** Vanessa Troiani, Chase C. Dougherty, Andrew M. Michael, Ingrid R. Olson

**Affiliations:** ^1^Department of Psychology, Temple UniversityPhiladelphia, PA, USA; ^2^Geisinger-Bucknell Autism and Developmental Medicine InstituteLewisburg, PA, USA

**Keywords:** fMRI, visual processing, individual differences, motivated attention, vision

## Abstract

Face processing involves a complex, multimodal brain network. While visual-perceptual face patches in posterior parts of the brain have been studied for over a decade, the existence and properties of face-selective regions in orbitofrontal cortex (OFC) is a relatively new area of research. While regions of OFC are implicated in the emotional processing of faces, this is typically interpreted as a domain-general response to affective value rather than a face- or socially-specific response. However, electrophysiology studies in monkeys have identified neurons in OFC that respond more to faces than any other stimuli. Here, we characterize the prevalence and location of OFC face-selective regions in 20 healthy college students. We did this by including another biologically motivating category (appetizing foods) in a variant of the standard face localizer. Results show that face-selective patches can be identified at the individual level. Furthermore, in both a region of interest (ROI) and a whole brain analysis, medial regions of the OFC were face-selective, while lateral regions were responsive to faces and foods, indicating a domain-general response in lateral OFC. Medial OFC (mOFC) response to faces scales in relationship to a measure of social motivation that is distinct from face processing abilities associated with fusiform cortex.

## Introduction

Social animals have evolved neural mechanisms that cause them to preferentially orient towards stimuli with social value, as well as seeking out social interactions because they find them rewarding. Evidence for this is found in studies showing that we unconsciously orient our eyes and attention in the direction indicated by another’s eye gaze (Frischen et al., 2007), and that we visually track individuals of higher social status, either because they are threatening or because they are rewarding (Foulsham et al., 2010). Smiles are considered sexually attractive (Otta et al., 1996) and are treated as a reward by primitive neural structures that process basic rewards such as food (Tsukiura and Cabeza, 2008). Moreover there is evidence from health psychology that social interactions make us feel better physically while the lack of social interactions and social support makes us depressed, lonely, and shortens our lives (Holt-Lunstad et al., 2015). These findings suggest that, we have evolved neural mechanisms for finding conspecifics interesting, essential, and rewarding (Chevallier et al., 2012).

Research on the neural basis of face processing has outlined a network of regions extending from the occipital lobe to the anterior temporal lobes (ATLs) involved in different aspects of face processing. Regions included in the face-processing network include nodes in the ventral visual stream such as the fusiform face area (FFA), and ventral ATL, as well as a region in the posterior superior temporal sulcus, and the amygdala (AMY). The combinatorial activity of these regions allows one to retrieve a vast array of information about any given individual, from identity and biographical details to affective and reward information. The latter type of information may rely on a newly discovered face patch found in both the human and macaque orbitofrontal cortex (OFC; Tsao et al., 2008a,b; Rajimehr et al., 2009). A detailed functional description of this region is lacking, although some findings indicate that portions of the OFC are involved in processing the emotional aspects of faces, including emotional valence and facial attractiveness (Aharon et al., 2001; Ishai, 2007; Cloutier et al., 2008; Chatterjee et al., 2009; Liang et al., 2010). Recent work by Pegors et al. (2015) has provided evidence for a face-specific value signals in human OFC as activity in this region titrated with the physical attractiveness of faces but not that of non-social, scenic stimuli. However, this study does not allow us to ascertain whether the OFC face patch responds more to faces or whether it responds to all stimuli of high reward value since scenic stimuli are not typically considered rewarding and do not have biological relevance.

This question is pertinent because a popular view of the OFC is that it serves a domain-general role in valuation such that value computations in the OFC are abstracted across stimulus categories. This is typically referred to as a “common currency” response in the OFC (Levy and Glimcher, 2012). Cells in the macaque OFC are responsive to primary rewards, such as food (Kringelbach et al., 2003) and in humans, multiple studies examining brain responses to food have found increased activation in OFC when participants look at high fat food pictures compared to low fat food pictures (Killgore et al., 2003). Activations in OFC are increased in human subjects following a period of fasting, which is interpreted as a heightened response due to the increased value of the food following to the participant’s relative state change (Page et al., 2011; Goldstone et al., 2014). Portions of the OFC are also responsive to abstract rewards, such as money (Knutson et al., 2001, 2003; Kim et al., 2011). The common currency view is at odds with the alternative view that there are subregions of the OFC that are particularly sensitive to social rewards.

In this study, we tested the hypothesis that the functional role of OFC face patches in the greater face network is to process social reward value. The more general goal of the current study was to provide a detailed investigation of the OFC face patch. Here we address the following questions: (1) In which subregions of the OFC are face patches located and in what percentage of participant’s can they be identified? Previous fMRI work in non-human primates has identified these patches in the lateral orbital sulcus (Tsao et al., 2008b). Because the OFC can be parsed into subregions, particularly medial and lateral segments, we visually inspected medial and lateral portions individually and within an region of interest (ROI) analysis to better describe their precise location. (2) Are there gender differences in OFC face patches? This analysis was motivated by the well-established finding that females tend to out-perform males on many tests of social functioning (Hall, 1978; Lewin and Herlitz, 2002). (3) Are OFC face patches sensitive to all rewarding stimuli or just socially rewarding stimuli? This question is motivated by work that has found OFC activation to other rewarding stimuli, such as food (Killgore et al., 2003; Kringelbach et al., 2003; Beaver et al., 2006). Because faces tend to be the most inherently rewarding stimulus used within “classic” face localizer tasks, previous OFC activation to faces could be face-specific or reward-specific. (4) Our hypothesis that the OFC face patch plays a key role in processing affective and motivational information about conspecifics predicts that this region should have clinical relevance for social behavior. Does activity in the OFC face patch predict interest or enjoyment in social interactions? Last and relatedly, (5) Is the OFC face patch functionally connected with other regions in the broader face network?

To examine these questions, normal college-aged participants underwent a standard fMRI scan while viewing blocks of stimuli of various object categories, including categories that have biological relevance and reward value (faces, food) and those that do not (places, objects). In addition, all participants completed a resting-state scan immediately preceding the localizer task.

## Materials and Methods

### Participants

Twenty right-handed adults [10 females; ages 21–31 years; mean = 24.0 (±2.6)] with normal or corrected-to-normal vision were recruited from Temple University and the surrounding community. Education level of these participants was quite high, with an average education equivalent to a 4-year bachelors degree [16 years of education (±1.4)]. All participants gave written informed consent in accordance with procedures approved by the Temple University Institutional Review Board and were paid for their participation. Participation consisted of an fMRI scan in addition to one 2-h behavioral session where questionnaires and experimental behavioral measures were collected, including those reported here.

### Behavioral Measures

Questionnaire measures acquired at the behavioral session included:

- (1) Broader Autism Phenotype Questionnaire (BAP-Q; Hurley et al., 2007). Participants completed the BAPQ, a freely available, 36-item self-report measure reporting on social aloofness, rigidity, and pragmatic language. Questions are rated on a 6-point scale from “very rarely” to “very often”. Best estimate scores were calculated by averaging informant report scores. Higher scores indicate higher loading on autistic traits. We were specifically interested in the social aloofness subscale, in order to examine the role of social motivation as a predictor of OFC face patch presence and response. Average scores for the aloof subscale and other subscales were as follows: Aloof, 3.23 (±0.28); Pragmatic Language, 3.26 (±0.46); Rigidity, 3.48 (±0.43).
- (2) Body Mass Index (BMI) was used as an index of food motivation. Participants reported their height and weight, which was used to calculate BMI. Average BMI for the group was 24.3 (±3.7) which is in the high normal-healthy range. Although BMI is not classically used as an index of food motivation, we chose this metric to serve as a trait-level variable to match our trait-level social motivation metric (BAP-Q Aloof subscale).
- (3) Hunger Rating. Just prior to the scan, participants were asked to rate their current hunger level on a scale from 1 to 10 where 1 is not hungry at all and 10 is extremely hungry. Participants’ average hunger rating was 3.25 on this scale (±2.1), indicating that they were only slightly hungry.

### Experiment Task and Stimuli

Stimuli were full color, 400 × 400 pixel images that included 80 faces, 80 scenes, 80 high fat foods, and 80 clocks. Images were acquired via google image searches or from previous in, house databases of stimuli used in published work (Troiani et al., 2014). Faces included equal numbers of male and female faces, as well as an equal number of famous and unknown, but attractive, faces, with images cropped such that the face took up the majority of the image, but facing direction was not limited to a certain angle. Places consisted of indoor and outdoor, natural and manmade scenes. Foods contained images of appetizing foods. Clocks were chosen in order to have a contrasting control object category that is typical for localizing face-selective regions. It is standard to have an object category that serves as a visual-perceptual control condition in order to isolate face-selective regions of cortex. However, we did not want the control category to be more interesting to subjects simply because of a wider variety of stimuli present within this category. Therefore, we chose the category of clocks because of the large variety of clock exemplars available and the ability to recognize clocks from multiple viewing angles. Clock images contained both digital and analog clocks from various angles. All 320 images were individually rated for hedonic value by an independent set of 20 raters using a 9-point likert scale [Like Extremely (9), Like Very Much (8), Like Moderately (7), Like Slightly (6), Neither Like nor Dislike (5), Dislike Slightly (4), Dislike Moderately (3), Dislike Very Much (2), Dislike Extremely (1)]. Mean values (± standard deviation) for each category were Clocks [4.9 (1.1)]; Places [5.8 (1.1)]; Faces [6.2 (0.94)]; Food [6.8 (1.1)]. Intraclass correlation coefficients (ICC) showed moderate to strong agreement between raters (ICC = 0.67). We find a significant difference between the two “reward” categories (faces, food) relative to our control categories (places, clocks; *F* = 30.9, *p* < 0.001). More specifically, raters preferred Faces relative to Places (*t* = 2.13, *p* = 0.047) and Clocks (*t* = 6.61, *p* < 0.001), Food relative to all other object categories (Places; *t* = 3.32, *p* = 0.004; Faces; *t* = 2.60, *p* = 0.018; Clocks; *t* = 6.42, *p* < 0.001), and Faces, Food, and Places over Clocks (all *t*’s > 5, all *p*’s < 0.001). Thus, we do not anticipate that any difference between Faces and Food would be due to the stimuli in our Face category merely having a higher hedonic value relative to our other reward category, Food, since raters generally liked the food images the most.

**Figure 1:**
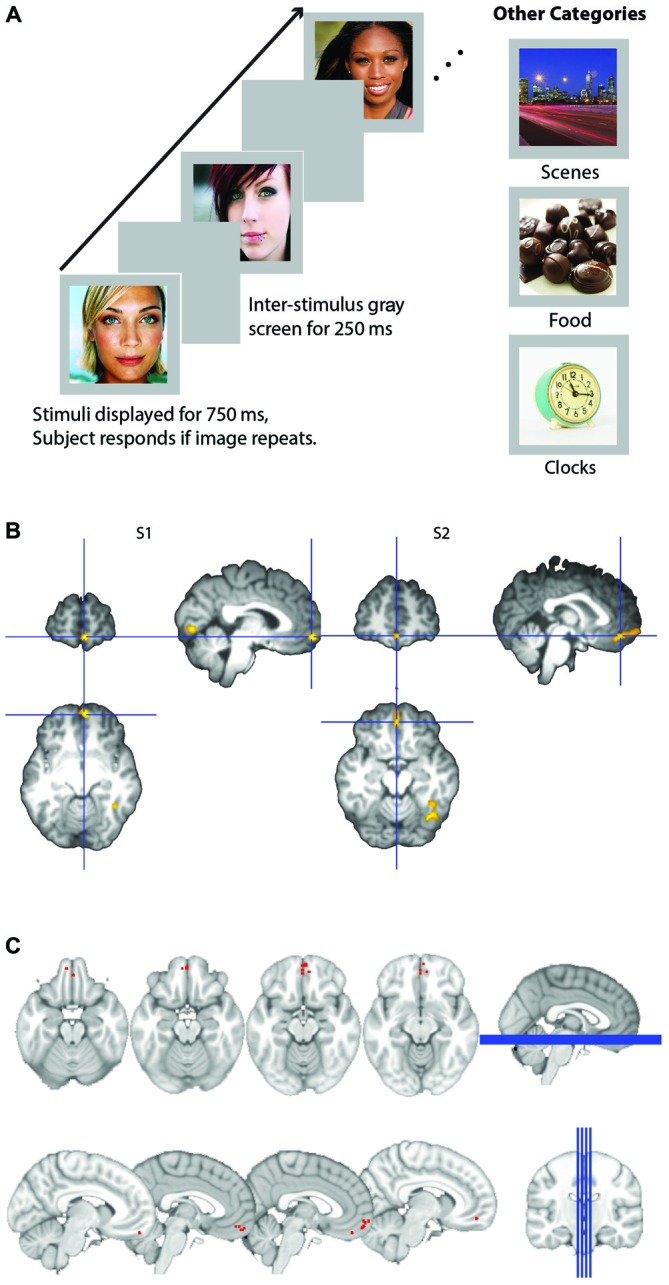
**(A)** Schematic of experimental design. Participants viewed images presented for 750 ms within a block and detected images that were consecutive repeats. Images were either male or female attractive and/or famous faces, scenes, appetizing food images, or clocks. **(B)** Representative subjects’ orbitofrontal cortex (OFC) face patch. Activation in medial OFC (mOFC) for the contrast of face stimuli compared to all other objects for two representative subjects. Contrast map is projected onto each subject’s high resolution T1, after skull extraction. OFC patches are located at the intersection of the blue crosshairs. **(C)** Plot of loci of all 14 subjects medial face patches. Loci are depicted as the coordinates of each subject’s peak voxel that responded more to faces than any other stimulus in the mOFC.

Responses to all stimuli were recorded with a five-key fiber optic response in the shape of the hand. Participants responded with their right index finger whenever they detected an image that appeared twice in a row, consecutively. This task was chosen to ensure that participants were paying attention to all stimuli. Experimental presentation was done with E-Prime. Images were presented in a continuous stream, each appearing on the screen for 750 ms, separated by 250 ms of a blank screen (see Figure 1A). Eight images of each category were randomly presented in each block with two consecutive stimulus repeats (total of 10 images in a block). Blocks were presented in randomized “super-blocks” that consisted of two blocks of each category (faces, places, food, and clocks), along with two blocks of rest (10 s each block). Superblocks were randomized, such that the presentation of the different categories was unpredictable. Each run consisted of four superblocks, resulting in a total of 80 face images, 80 places images 80 food images, and 80 clock images presented during each run (64 unique stimuli and 16 repeats per category). These images were then repeated for the three subsequent runs, each with a unique presentation order of superblocks. All participants were presented with the same run and category orders, but within a category, image selection was completely randomized for each participant. Each of the four runs was 7.1 min long (142 volumes acquired with a TR of 3 s = 426 s). Four acquisitions were acquired prior to the start of stimulus presentation to allow for T2 stabilization and four acquisitions were acquired following the end of stimulus presentation to capture the full HRF for the final block.

### Magnetic Resonance Imaging Acquisition

Imaging data were collected using a 3T Siemens Verio scanner and a 12-channel head coil. A high-resolution T1-weighted MPRAGE sequence of the entire brain (176 sagittal slices, isotropic voxel size = 1 mm, repetition time (TR) = 1900 ms, echo time (TE) = 2.54 ms, flip angle = 9°) was acquired for the registration of fMRI data to standard space. The T1-weighted images were acquired using a three-dimensional magnetization prepared rapid acquisition gradient echo pulse sequence. Functional data consisted of one 6-min run of resting state and four 8-min runs of whole-brain T2* weighted BOLD echoplanar images with 142 volumes acquired per run (61 oblique axial slices, 2.5 mm slice thickness, voxel size = 3 × 3 × 2.5 mm; matrix size = 80 × 80; TR = 3000 ms, TE = 20 ms, flip angle = 90°, GRAPPA = 2). We optimized for signal in the OFC by tilting slice acquisition −30° from the AC-PC plane reduce signal dropout (Deichmann et al., 2003).

All runs were estimated for movement. For the main experimental task, one participant moved greater than 5 mm in one run; data from this run was not included in subsequent analyses. After removal of this run, absolute movement across participants averaged 0.23 mm (±0.16 mm). Movement during the resting state run averaged 0.16 mm (±0.09 mm) across subjects. One subject’s resting state data was erroneously acquired using different slice acquisition parameters than the rest of the group. This subject’s data were not included in the resting state analyses.

### Data Analysis

The data analysis consisted of three parts: (1) the localization of individual face patches based on whole brain analyses; (2) ROI analysis of the main experimental task (adapted face localizer) based on masks defined by the Automated Anatomical Labeling (AAL; Tzourio-Mazoyer et al., 2002) atlas in OFC and other reward/face processing regions; and (3) functional connectivity analyses of resting state data using subject-specific ROIs obtained from individually-defined face-selective peaks.

#### Face/Food Localizer

Image preprocessing and statistical analyses were performed using FSL. Data preprocessing and univariate analysis of fMRI data were performed using FEAT (fMRI Expert Analysis Tool) version 6.0, part of the software library of the Oxford Centre for functional magnetic resonance imaging of the brain (fMRIB).^1^

Gradient field maps were used to correct for distortion due to field inhomogeneities. Functional images from both experimental and localizer scan runs were initially analyzed separately for each participant. Preprocessing steps included non-brain removal using BET, motion correction using MCFLIRT, high pass temporal filtering with a 100 s cutoff, and undistorting of the EPI data to correct for magnetic field distortions using individual field maps. EPI data was then registered to each participant’s T1-weighted anatomical scan using BBR. The data were spatially smoothed with a 5 mm FWHM isotropic Gaussian kernel.

Preprocessed data were then submitted to a standard general linear model. These models included four categorical regressors indicating whether the stimulus for each block was a face, place, food, or clock. Categorical regressors were boxcar functions at stimulus onset convolved with a canonical hemodynamic response function.

#### Subject-Specific Analysis: Characterization and Localization of Face-Selective Responses in OFC

We examined whole-brain maps for each individual subject, in order to examine whether face-selective nodes in the OFC could be detected on an individual level, similarly to perceptual face processing nodes (e.g., FFA). To do this, each subject’s statistical maps for the contrast of Faces > Places, Food, and Clocks was viewed at a t score threshold of 2.0. If a cluster of more than five contiguous voxels could be identified at this threshold, peak coordinates of these activations were recorded. Boundaries for each search were based on previously reported guidelines and anatomical landmarks.

We then identified subject-specific peaks within face-selective nodes by searching for clusters of activation above a threshold of *T* > 2.0, within anatomical boundaries. This threshold was chosen based on previous work identifying individually-defined, category-specific responses in the brain (Vass and Epstein, 2013; Troiani et al., 2014). The FFA was defined as the region within the fusiform gyrus, between the collateral sulcus and the temporo-occipital sulcus that was more active for faces compared to all other objects. The AMY was defined as the region in anterior temporal cortex with a superior boundary of the mammillary bodies, a medial boundary of the uncus and a posterior boundary of the temporal horn of the lateral ventricle (superior) and hippocampus (inferior). Definitions of two anterior temporal clusters were defined based on previous observations in our lab and others of two distinct anterior temporal patches: the first appears in perirhinal cortex and is referenced as Anterior Face Patch 1 (AP1) by Tsao et al. (2008a). The second appears at the anterior-most tip of the temporal pole and is referenced as Anterior Face Patch 2 (AP2). OFC clusters were defined as peaks within the region of ventral frontal cortex including the gyrus rectus, and medial, anterior, posterior, and lateral orbital gyri, within the superior boundary of the corpus callosum. Clusters with a peak within gyrus rectus were labeled “medial”, while clusters within medial, anterior, or lateral orbital gyri were labeled “lateral”. We did not observe any peaks within the posterior orbital gyrus.

#### Group Analysis: Region of Interest Based on Atlas-Derived Masks

We also performed a ROI analysis using regional definitions from the AAL atlas. We chose these regions to characterize medial and lateral OFC response to faces, as well as to establish activation patterns in nearby control regions known to demonstrate face-sensitivity (AMY) and general reward responsive regions (nucleus accumbens). The AAL atlas has four separate ROIs that span the OFC (described as lateral left, lateral right, medial, and subcallosal in the atlas). The two medial ROIs (medial and subcallosal) refer to anterior and posterior parts of medial OFC (mOFC), respectively. Although we were primarily interested in medial-lateral differences, we report results for all four ROIs and refer to analyses using the two medial AAL OFC ROIs as anterior-medial (named mOFC in AAL atlas) OFC and posterior-mOFC (named subcallosal OFC in AAL atlas), so as not to confuse this with more general discussions of medial vs. lateral OFC.

For individual parameter estimate ROI analyses, the time course of response during the main experiment was extracted from each ROI and response estimates (i.e., Beta values) were obtained for each regressor and covariate, which were then compared between conditions using a repeated-measures ANOVA with follow-up *t*-tests, when appropriate.

#### Group Analysis: Whole Brain

Individual whole brain maps were submitted to a second-level *t*-test for the contrast of face stimuli compared to all other stimuli. To control for multiple comparisons, we used threshold-free cluster enhancement (TFCE; Smith and Nichols, 2009), which determines statistical significance using permutation labeling, with the *α* level set at *P* < 0.05.

#### Resting State Analyses

In addition to task-based analyses, we also analyzed data acquired from a 6 min resting state study, where participants were instructed to keep their eyes on a plus sign for the first 6 min of the scanning session. BOLD data were simultaneously acquired. Data were then brain extracted, motion corrected, slice-time corrected, spatially smoothed (5 mm FWHM), undistorted, *z*-normalized, and co-registered to each participant’s T1-weighted anatomical scan and subsequently re-sampled to the standard 2 mm MNI-152 template. Region-specific nodes were identified by individually identifying the peaks showing greater activity for faces greater than all other objects from the adapted face localizer (anatomical definitions described in detail above). For those individuals who did not have a particular node, mean coordinates from those individuals with that region were used. Spheres of 9 mm radius were generated, centered on the voxel with the highest activation within each peak. These were then used as masks for the pair-wise resting state analysis.

Although we did not anticipate motion-related artifacts with this population of subjects, we took several measures to reduce the impact of motion related artifact via scrubbing techniques. The six motion parameters identified for each subject were modeled as nuisance regressors and included in our model. Average signal time course from lateral ventricles and white matter were estimated prior to spatial smoothing and specified as nuisance regressors. A bandpass filter was then applied to remove high-frequency fluctuations or noise associated with non gray-matter tissue back into the RS data (Carp, 2013; Jo et al., 2013; Power et al., 2013).

It should be noted that we chose not to perform global signal regression (GSR), as it has been shown to exacerbate distance-dependent motion related biases (Jo et al., 2013) and to be of limited efficacy when combined with comprehensive data censoring approaches (Yan et al., 2013). Furthermore, the inclusion of GSR can artificially increase the number of negative correlations seen in resting state data (Murphy et al., 2009; Weissenbacher et al., 2009; Saad et al., 2012). *z*-normalization has been shown to similarly reduce motion-induced artifacts in RS data without inducing artificial negative correlations (Yan et al., 2013). Partial correlations between mOFC face patches and other regions in the face network were generated in order to determine network connectivity during resting state. All *p*-values were Bonferroni corrected for multiple comparisons. One of these individuals had an error in their resting state data acquisition (noted in the methods, previously), reducing the N for this portion of the analysis to 19.

## Results

### Questions 1: Where are OFC Face Patches Located and in What Percentage of Participant’s Can they be Identified?

We quantified the presence of face network nodes by identifying the presence or absence of each face-processing region in each of the 20 subjects. Regions included bilateral FFA, bilateral AMY, bilateral temporal pole, bilateral medial anterior temporal patches, and finally, medial and lateral OFC. We did not include the posterior superior temporal sulcus because this region is primary implicated in processing dynamic aspects of faces (Allison et al., 2000). Table 1 tabulates the number of participants with OFC face patches. Similar to previous reports (Tsao et al., 2008a), only a subset of participants had OFC face patches.

**Table 1:** **Number of participants out of 20 with each node of the face network**.

	Number of subjects with each face network node		
	Left	Right
FFA	17	19
Amygdala	14	14
Temporal pole	10	10
Medial ATL	9	13
Lateral OFC	13	13
Medial OFC		14

*Seventy percent of participants had a medial OFC face patch, similar to the number that had temporal pole or medial anterior temporal lobe face patches*.

OFC face patch coordinates are listed in Table 2. There was a great deal of variability in the location of these patches, presumably due to the known large inter-individual differences in gyral and sulcal morphology found in the OFC (Chiavaras and Petrides, 2000; Kringelbach, 2005). The patches were most consistently found in mOFC (see Figure 1B for representative subjects and Figure 1C for a plot illustrating the peak value of medial face patches in all 14 subjects with a patch), with lateral patches observed in most individuals that had medial patches. However, one individual did have lateral patches but no medial patch. For comparison, we also present the portion of individuals who have other nodes in the face-processing network.

**Table 2:** **Coordinates of OFC face patches in 20 participants**.

	Lateral OFC						Medial OFC		
	Left			Right		
Participant	*X*	*Y*	*Z*	*X*	*Y*	*Z*	*X*	*Y*	*Z*
1
2	−30	42	−16	34	44	−8	4	60	−10
3	−38	24	−22	42	50	−14	0	46	−14
4	−20	48	−12	36	36	−6	2	58	−8
5
6	−34	54	−6				4	56	−16
7	−38	24	−16	28	36	−18	0	56	−16
8	−38	32	−16	28	30	−16	−4	54	−8
9	−26	28	−18	28	32	−18	2	64	−8
10	−38	32	−14	32	40	−8	0	54	−18
11	−38	34	−14	24	34	−14	0	50	−14
12
13	−44	34	−18	24	36	−12	0	52	−18
14	−26	26	−18	24	32	−14	−6	52	−20
15				36	46	−12	4	58	−14
16	−36	24	−22				2	44	−22
17	−28	40	−10	44	46	−12
18
19				22	50	−12	8	48	−14
20

### Question 2: Are there Gender Differences in OFC Face Patches?

We investigated whether a participant’s gender impacted the presence of OFC face patches in two ways. We first quantified whether each individual had an OFC face patch present in the subject-specific characterization analysis. Presence of medial or lateral OFC face patches were not significantly greater in one gender compared to the other (all *p*’s > 0.3). Next, we looked for differences in activation of OFC subregions, as defined by the atlas-based ROI analysis. In a repeated measures ANOVA with gender as a covariate, there was no evidence of a region × gender or condition × gender interaction (all *p*’s > 0.5).

### Question 3: Are OFC Face Patches Sensitive to All Rewarding Stimuli or Just Socially Rewarding Stimuli?

For this question, we utilized OFC masks from the AAL atlas in order to independently assess subregion activation. *Face selectivity*, which was defined as greater activations to faces as compared to primary rewards (e.g., food), was only found in the anterior-medial and posterior-mOFC ROIs. Lateral OFC was significantly more responsive to rewarding objects in general, but was equally sensitive to social and non-social objects. Significant effects of region, condition, and region × condition interactions were found (Effect of Subregion; *F*_(8)_ = 56.7, *p* < 0.001; Effect of Category; *F*_(3)_ = 27.6, *p* < 0.001; Subregion × Category Interaction; *F*_(24)_ = 18.7, *p* < 0.001). Follow-up paired *t*-tests in each region were then completed to explore these effects. Posterior-mOFC was significantly more responsive to faces compared places (*t* = 3.57, *p* = 0.002), food (*t* = 2.56, *p* = 0.019), and clocks (*t* = 3.15, *p* = 0.005). Anterior-mOFC showed a similar face-selective response, with significantly greater activation to faces compared to all other stimulus types (All *t*’s > 3.7, All *p*’s < 0.001). The left lateral OFC was more responsive to rewarding objects compared to non-rewarding objects (faces-places; *t* = 4.25, *p* < 0.001; faces-clocks, *t* = 4.74, *p* < 0.001; food-places; *t* = 3.811, *p* = 0.001; food-clocks; *t* = 4.14, *p* = 0.001), but equally responsive to faces and food (ns, *t* = 0.644, *p* = 0.527). This pattern of results was similar in the right lateral OFC, except for a non-significant difference between food and clocks (faces-places; *t* = 4.69, *p* < 0.001; faces-clocks, *t* = 3.10, *p* = 0.006; food-places; *t* = 3.40, *p* = 0.003; food-clocks; ns, *t* = 0.89, *p* = 0.386; faces-food; ns, *t* = 0.644, *p* = 0.386).

**Figure 2:**
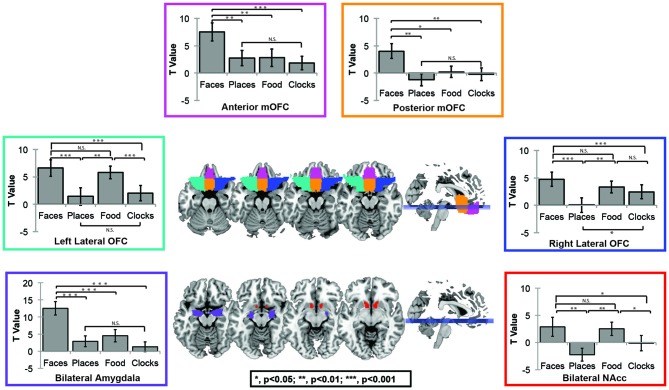
**Automated Anatomical Labeling (AAL) Atlas region of interest (ROI) Figure.** The AAL atlas was used to define masks for four OFC subregions including left (green) and right (blue)lateral OFC, posterior mOFC (orange), and anterior mOFC (pink), bilateral AMY (purple) and bilateral nucleus accumbens (red).

For comparison sake, we present the sensitivity profile of OFC ROIs, nucleus accumbens, and AMY ROIs in Figure 2. As expected, the nucleus accumbens was sensitive to rewarding objects compared to non-rewarding objects (All *t*s > 2.0, all *p*s < 0.05), but did not differentiate between faces and food (ns, *t*_(19)_ = 0.23, *p* = 0.823), while the AMY was more sensitive to faces than all other objects (All *t*’s > 4.8, all *p*’s < 0.001).

**Figure 3:**
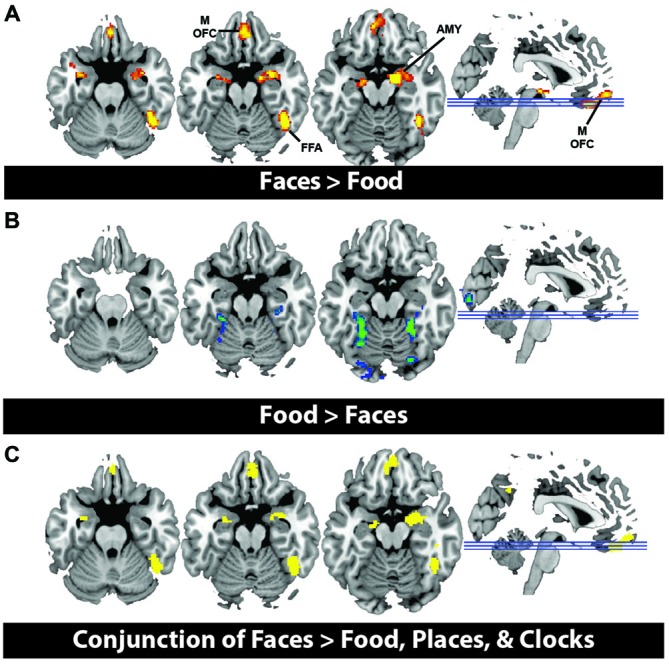
**(A)** Activation from whole brain group analysis in 20 subjects. **(A)** Contrast of Face Stimuli > Food Stimuli. Significant peaks are labeled, including mOFC, fusiform face area (FFA), and amygdala (AMY). **(B)** Contrast of Food Stimuli > Face Stimuli. This illustrates no significant regions of activation in OFC for food relative to faces. All images are thresholded at *p* < 0.05, cluster corrected for multiple comparisons. **(C)** Conjunction of Faces > Food, Places and Clocks. This is a binary rendering that depicts voxels that were significantly activated in the separate contrasts of Faces > Food, Faces > Places, and Faces > Clocks, illustrating that these regions responded more to face stimuli than any other type of object.

In order to illustrate that this face-selective effect was not merely due to the use of a ROI based approach, in which activations across many voxels are averaged, we also present results at the whole brain level. In a direct contrast of Faces compared to Food, we find greater activation in the mOFC, AMY, and right fusiform, consistent with our ROI analysis (Figure 3A, Table 3). The spatial extent of this mOFC activation spanned regions that are spatially included in both medial-lateral and posterior-lateral ROIs from the previous analysis. In contrast, we show that the only regions that respond more to Food than Faces are in regions of early visual cortex (Figure 3B, Table 3). When contrasting Faces or Food with our two control stimulus sets (Places and Clocks), we find activation in lateral OFC and the nucleus accumbens for both contrasts. This indicates that both Food and Faces activate regions involved in reward processing (and again, is consistent with our ROI analysis). Finally, in order to illustrate that this response was due to face-selectivity, we present a conjunction of Faces > Food, Places, and Clocks. To derive this map, significant voxels from each of the individual contrasts, Faces > Food, Faces > Places, and Faces > Clocks, were computed. The conjunction of these three contrasts was then computed by selecting voxels that were significant in all three of these individual contrasts. Again, we find that faces selectively activated mOFC, AMY, and the right FFA (Figure 3C). There were no regions of the OFC that activated more for non-rewarding (places, clocks) relative to rewarding (faces, food) stimuli. Places relative to all other objects activated the expected regions of bilateral parahippocampal place area, retrosplenial cortex, and the transverse occipital sulcus. Clocks relative to all other objects activated the expected region of bilateral lateral occipital cortex, a region known for its involvement in object processing.

**Table 3:** **Coordinates of whole brain analysis contrasts**.

	Coordinates			Maximum *z* value	# of voxels	Anatomical region
Contrast	*X*	*Y*	*Z*			
Faces > Food	64	−50	8	4.97	1067	Right middle temporal gyrus
	18	−4	−16	5.71	986	Right amygdala
	28	−6	−34	4.44	(subpeak)	Right anterior temporal face patch
	44	22	30	4.69	432	Right middle frontal gyrus
	0	42	−24	4.21	344	Orbitofrontal cortex
	46	−52	−20	5.48	288	Right temporal occipital fusiform cortex (Fusiform face area)
Food > Faces	24	−44	−12	5.87	5782	Right lingual gyrus
	−44	−48	−6	4.55	345	Left inferior temporal gyrus

### Question 4: Does Activity in the OFC Face Patch Predict Interest or Enjoyment in Social Interactions?

**Figure 4:**
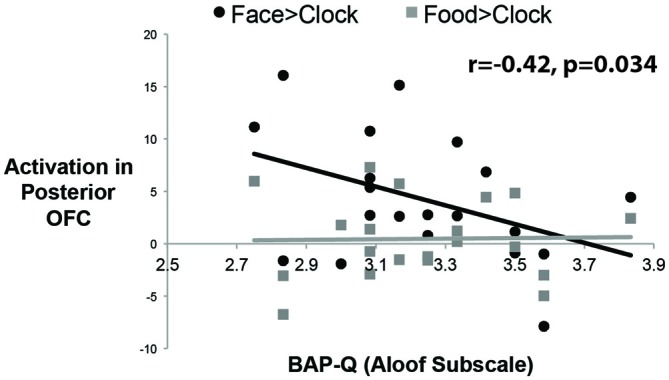
**Correlations between activity in the posterior OFC and social interest/motivation as measured by the Broad Autism Phenotype Questionnaire (BAP-Q).** Each black dot represents the data from a single subject for the social reward contrast, Faces vs. Control Objects; each gray dot represents the data from a single subject for the non-social reward contrast, Food vs. Control Objects. The correlation was significant only for the face contrast.

To answer this question, we measured social enjoyment and motivation by the Aloof subscale of the BAP-Q. This subscale asks questions that index how much one seeks social interactions and enjoys them. We then correlated scores on this measure with face- and food-selective activation levels in both mOFC ROIs using the contrast of Faces or Food relative to Clocks (see Figure 4). There was a significant correlation between face-selective activity in posterior-mOFC and aloofness, such that higher activations predicted higher social interest and motivation (*r* = −0.42; *p* = 0.034). This relationship does not appear to be driven by general insensitivity to rewarding stimuli in aloof individuals as there was no relationship between social aloofness and activations to rewarding food stimuli (food vs. clocks; *r* = 0.020, *p* = 0.43). Interestingly, a different individual difference variable predicted neural activity to rewarding food stimuli: BMI. When we correlated these same face- and food-selective responses with BMI, increased BMI corresponding to an increased response to food but not faces (relative to clocks) in the anterior-mOFC (anterior-mOFC, *r* = −0.38, *p* = 0.048; posterior-mOFC, n.s., *r* = 0.043, *p* = 0.429).

### Question 5: Is the OFC Face Patch Functionally Connected with Both the Greater Face Network at Rest?

**Figure 5:**
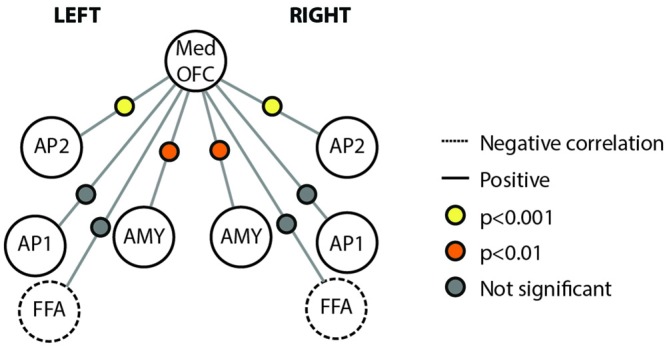
**Connectivity schematic depicting mOFC Face Patch connectivity with other face network nodes.** Positive correlations depicted with solid lines, negative correlations depicted with dotted lines. Significance of correlation across subjects for each connection depicted by colored dot between the two relevant nodes. Yellow indicates *p* < 0.001; Orange indicates *p* < 0.01; gray indicates not significant. Structures included in the figure are Med OFC, medial OFC; AP2, Anterior Temporal Face Patch 2; AP1, Anterior Temporal Face Patch 1; AMY, Amygdala; FFA, Fusiform Face Area.

We also examined the connectivity between nodes of the face processing network outside of a face processing task using a resting state experiment that was acquired prior to our localizer task. We found reliable pair-wise partial correlations between the mOFC face patch (defined based on individual subject peaks) and bilateral anterior temporal face patches (AP2; left; *r* = 0.48, *p* < 0.001; right; *r* = 0.39; *p* < 0.001) and bilateral AMY (left; *r* = 0.26, *p* = 0.003; right; *r* = 0.23, *p* = 0.007). Connections between mOFC and right FFA approached significance (*r* = −0.11, *p* = 0.085), but did not reach significance in left FFA (*r* = −0.10, *p* = 0.348) or bilateral medial APs (perirhinal/AP1; left; *r* = 0.10, *p* = 0.627; right; *r* = 0.09; *p* = 0.762). The connectivity results from the resting state analysis are visually depicted in Figure 5.

## Discussion

The goal of the current study was to provide a detailed investigation of putative face patches in the human OFC. This investigation was motivated by research in macaques showing the presence of clusters of face sensitive neurons in the OFC (Tsao et al., 2008a,b; Rajimehr et al., 2009). We hypothesized that the functional role of OFC face patches in the extended face network is to process and evaluate social reward value. Our design and analysis allowed us to address this question in addition to other distinct questions about OFC face patches.

Our results show that OFC face patches can be reliably identified using an adapted face localizer task in 70% of participants. This is somewhat higher than the number of humans with frontal face patches reported in other studies (Tsao et al., 2008a). We may have been more successful in isolating this region due to our utilization of optimized acquisition parameters that included a low echo time and tilted slice acquisition. There was a great deal of variability in the location of these patches, consistent with the highly variable structural morphology of the greater OFC (Kringelbach, 2005).

### Significance of Findings For Understanding Social Reward Processing

There is a population of neurons in the macaque OFC with response profiles to faces that are similar to that found in neurons recorded from in the ventral visual stream. These neurons were first described by Thorpe et al. (1983) but were subsequently reported by other investigators (Thorpe et al., 1983; Booth and Rolls, 1998; Rolls et al., 1999; Rolls, 2000a). Rolls and Baylis (1986) reported that these neurons were harder to activate then temporal cortex face-selective cells, that they responded with relatively longer latencies (130–220 ms), and that they responded better to real faces than pictures of faces. Rolls (2000b) speculated that the function of these face neurons was primarily in social learning since specific faces might be associated with other information that can act as a reinforcer (e.g., a previously violent attack or a pleasant touch). More recently, Watson and Platt (2012) showed that a greater number of neurons in the primate OFC signaled social category than fluid value, indicating a preference of neurons in this region for social computations. We find that this face-selectivity can also be identified using the resolution available with fMRI. Furthermore, our findings are consistent with a variant of Rolls’ view of the OFC’s role in social learning since we show a relationship between trait-levels of social interest and activations to faces in the posterior-mOFC.

Our finding that OFC face patches are related to a domain of social motivation that is relevant to autism spectrum disorders (ASD) offers the intriguing possibility that variable responses in this region reflect behavioral adaptations that are related to neuropsychiatric symptoms. It is well known that children with autism have altered reward sensitivity in behavioral tasks and that classic reward-responsive brain regions, like the nucleus accumbens, show atypical responses in this population (Scott-Van Zeeland et al., 2010; Delmonte et al., 2012; Kohls et al., 2014). While the OFC face patches have not been examined within the ASD population, the OFC has been implicated as a potential region of dysfunction in developmental disorders, including ASD. Morphometric differences have been found in the OFC of children and adults with ASD (Sawa et al., 2013), including reduced OFC gyrification and reductions of white and gray matter that correlated with social symptom severity (Hardan et al., 2006; Girgis et al., 2007). Functionally, OFC activation is reduced in ASD populations relative to age-matched peers for social reward learning tasks and self vs. other referential processing tasks (Lombardo et al., 2010). Even at rest, baseline connectivity between all nodes of the “social brain” are reduced in ASD relative to controls, particularly the OFC (Gotts et al., 2012). Thus, the OFC is a region that is strongly implicated as dysfunctional in ASD with regard to social processing, but future research should examine OFC face patches more specifically in individuals with ASD.

Beyond the OFC implication in ASD symptomology, this region has been linked at a more macro level to social and emotional behavior ever since Phineas Gage’s accident left him with an altered OFC and changed personality. Damage to the OFC typically leads to impulsivity, disinhibition, socially inappropriate behavior, and emotional changes. One patient population that is informative to the role of the OFC face patches in social cognition are those with behavioral variant frontotemporal dementia (bvFTD). This disorder is characterized by disinhibition, apathy/inertia, loss of sympathy/empathy, perseverative/compulsive behaviors, hyperorality, and a dysexecutive neuropsychological profile. bvFTD patients still seek out primary rewards (food, sex), but are impaired at learning and recognizing more complex stimulus-reward contingencies, such as those important for social interactions (Viskontas et al., 2007). The apathy observed in these patients has been linked to the atrophy of reward circuitry and is related to measures of social cognition (Eslinger et al., 2012). Thus, there does seem to be some level of social-specificity in terms of the role of OFC in these patients, although additional studies to probe the presence and role of face patches in their behavior is necessary and warranted. Although face processing is not widely studied in this population, one study found that FTD patients were impaired at recognizing facial expressions, but not facial identity, and that this impairment was also present in a vocal emotion recognition task, indicating domain general emotion recognition deficits (Keane et al., 2002). Another study found a specific impairment in assessing negative facial emotions in FTD patients (Fernandez-Duque and Black, 2005). Still others have found that FTD patients with primarily frontal atrophy have impairments in processing both negative and positive valence in faces, while those with primarily temporal atrophy only have difficulty with negative emotions (Rosen et al., 2004). Because these patients may also have damage or atrophy in fiber tracts that connect the OFC to other regions, it remains challenging to isolate the impact of OFC damage on social-emotional and face processing. Indeed, a large white matter tract called the uncinate fasciculus connects the AMY and ATLs to the OFC (reviewed in Von Der Heide et al., 2013) and is important for social network size (Hampton et al., 2016). In the resting state analysis in the current study, we find positive functional connectivity between the AMY and mOFC, which are linked by this particular tract. Thus, future work will need to consider both the integrity of individual brain regions as well as their connective tracts in social and motivational behavior. Regardless, there do appear to be social and face-processing deficits after OFC damage.

### Significance of Findings for Neuroeconomic Theories

We found an interesting medial/lateral split when comparing socially-rewarding stimuli (faces) to gustatory rewards (food images). mOFC regions were more responsive to pictures of faces than to pictures of appetizing food while lateral OFC was equally responsive to both social and non-social rewards. Likewise, the nucleus accumbens, a region that is critical for signaling reward value, was equally sensitive to both social and non-social rewards. This result is interesting and pertinent to discuss in the context of neuroeconomic theories. A popular idea in the neuroeconomics literature is that reward sensitivity in the OFC is primarily about an abstract and domain-general calculation of value. There are multiple findings showing that responses in the OFC to stimuli from multiple modalities (juice, money, sexually-relevant stimuli) are overlapping and that activity levels depend only on the value of the stimulus, rather than on the stimulus identity or category (Chib et al., 2009; Kim et al., 2011; Levy and Glimcher, 2012). Our results are contrary to this notion since we found some degree of selectivity. We note that single unit recording work in both the mouse and the monkey supports the existence of object/odor/taste- selective neurons in the OFC (Gottfried and Zelano, 2011). However, because these neurons tend to be intermixed with other object-selective neurons, it was unclear whether object-selectivity would be observed using the more course spatial resolution of fMRI, where populations of neurons would be averaged within a voxel. Our results confirm findings of face-selective nodes in human mOFC and suggest these patches can be resolved at the individual level in humans using fMRI techniques. We note that some regions that have a known role in computing value (lateral OFC and nucleus accumbens) showed equivalent response to both faces and food, with mOFC regions showing a face-selective response similar to that of another face-sensitive region, the AMY. From these results we infer that humans find other humans—even images of other humans—inherently rewarding, which likely serves as a catalyst for social interaction and cooperation.

The field of neuroeconomics has addressed spatially dissociated responses in OFC in terms of a anterior-posterior valuation gradient, with more primary rewards (erotic stimuli) encoded more posteriorly and secondary (learned, abstract rewards such as money) encoded more anteriorly (for recent review, see Sescousse et al., 2013). Our results do not necessarily support this valuation gradient, since face selectivity was more medial and lateral OFC was equally face- and food- selective. There are, however, critical differences in our task design compared to most of those used in neuroeconomics. Our task was a passive viewing task, where any difference between stimuli was due to spontaneous evaluation of the images. Typical neuroeconomics tasks require participants to make some sort of overt judgment or evaluation of the stimulus or utilize an incentive delay task where the participant must work for a potentially rewarding and variable outcome. For example, Pegors et al. (2015) found category-specific and overlapping value signals to beautiful faces and places, but only when the value dimension—beauty—was explicitly evaluated by participants during scan acquisition. In contrast, we evoked OFC activation for both food and social stimuli using a passive viewing task, where differences in OFC activation and relative value are merely due to spontaneous evaluation of the images, rather than a forced evaluation procedure. This suggests that reward-selective nodes can be isolated using short, block design tasks, rather than the much longer event-related designs typically required for incentive delay and valuation paradigms. The lack of an anterior-posterior valuation gradient suggests that task may be a critical factor in the emergence of such a gradient vs. a spatial topography of reward category selectivity in OFC.

Within the context of domain-general value and spatial gradients, it is also interesting to consider that we find somewhat specific social and food responses in anterior vs. posterior medial portions of the OFC. We found that individual differences in posterior-mOFC activations in response to faces correlated with self-reported social motivation, as measured by the Aloof subscale of the BAP-Q. In contrast, food-selective responses in anterior-mOFC increased as the BMI of individuals increased. Taste-responsive neurons exist in the macaque caudolateral OFC, a region with direct connectivity from the primary taste cortex in posterior insula (Rolls et al., 1990). A great deal of research on the macaque OFC has led to the conclusion that the taste sensitivity of this region is related to reward or value. For instance, hungry monkeys will work in order to get electrical stimulation of this brain region but a satiated monkey will not (Mora et al., 1979; Rolls et al., 1999). It is likely that there are both state (being hungry or satiated) and trait differences in how much one finds food rewarding. BMI may serve as a proxy variable for trait-level differences in intrinsic food-valuation. Our findings are consistent with prior findings that report neural activations in response to food cues in lateral OFC (Kringelbach et al., 2003; Beaver et al., 2006; Killgore and Yurgelun-Todd, 2006; Frank et al., 2010a,b; Burger and Stice, 2011). A smaller set of studies have reported changes in mOFC (Rolls and McCabe, 2007; McCabe et al., 2010) however it should be noted that in these studies, participants either subjectively rated each stimulus upon presentation and/or were in a fasted state. The relevance of task has been unexplored in this literature.

This is the first fMRI study to compare face and food categories, as these stimuli tend to be utilized be scientists studying separate fields of cognitive neuroscience. Although additional research is necessary, it is interesting to speculate as to why mOFC responds more to faces than to food overall, yet individual differences in trait-based food motivation reflect some level of scaled response to food in this same region. It is possible that this region was originally a domain-general relevance region and has evolved to become primarily responsive to social-relevance based on the need for social motivation in successful human interaction and survival. Perhaps individual differences in food relative to social motivation reflect individual differences in the adaptation of this relevance region, which is activated by faces in most individuals. Another option is that with increased food motivation (here assessed using BMI), this face-selective region can be “hijacked” in favor of other motivating categories. This speculation garners some support from studies that find social deficits in obese individuals (Baldaro et al., 2003) and additionally from an increased prevalence of obesity in autism (Curtin et al., 2010).

### Other fMRI Studies of OFC Response to Faces

Although this study represents the first comparison of social images to another highly rewarding stimulus (food images), previous fMRI studies have shown OFC activation in response to face images. However, this has primarily focused on relative differences in response to emotional faces rather than a categorical face response, *per se*. The OFC has been included as part of the extended face network, with the OFC involved in assessment of facial beauty and sexual relevance (Aharon et al., 2001; O’Doherty et al., 2003; Kranz and Ishai, 2006; Ishai, 2007). Indeed, the OFC responds in a gender- and sexual-orientation specific way, with more activation for the gender relevant to an individual’s sexual preference (Kranz and Ishai, 2006). However, because one can also interpret a sexual preference to be an individual difference in relative value, it remains unclear whether activation in this case is because of the increased value of the sexuality-specific stimulus or its pure sexual nature. Similarly, the activation of the OFC in face processing tasks may be merely due to the relative value of faces/social stimuli relative to other stimuli traditionally included as controls in these experiments (houses or scenes, objects, scrambled images). In the “Introduction” and earlier in the “Discussion” Sections, we mentioned the popular view that the OFC serves as a domain-general value computation region (Levy and Glimcher, 2012). Here, we show that the mOFC responds specifically to social stimuli, whereas lateral OFC responds equally to both food and social stimuli. Our findings are consistent with the history of animal literature that shows domain-specific neurons or nodes that respond to particular types of rewarding stimuli. Furthermore, we show that social aloofness, which is thought to be a stable trait, scales with activation in this region. Future work should examine whether these nodes are stable across changes in social and hunger states and relative to other categories of valuable stimuli.

## Conclusions and Future Directions

These results suggest that a region in the mOFC is dedicated to processing and automatically evaluating faces, potentially due to the importance of conspecifics to our well-being and survival as a social species. We show correlations with two metrics (BAP-Q and BMI) that are relatively stable over the course of weeks or months. Given the influence of state on OFC electrophysiological response profiles, future exploration of the influence of state-based changes in social and appetite-drive on face and food selectivity in these regions is warranted. For example, work from non-human primates has demonstrated changes in OFC response profiles of sensory-specific neurons in the OFC during various states. Neurons that are responsive to fat texture in the primate OFC reduce their response when fatty food is eaten. Thus, these neurons are both *stimulus-selective* and *change-modulated*, based on the current state of the organism. Studies in humans also demonstrate changes in OFC response to food stimuli following periods of fasting or satiation. Although change modulation in the OFC has only been reported in the food domain, the presence of face-selective OFC nodes suggests the intriguing possibility that face-selective regions in the OFC may also change responsivity based on the individual’s homeostasis. For example, if an individual is lonely or rejected, do these regions assist in motivating an individual to restores social homeostasis?

Furthermore, altered response properties in the OFC have been indicated in multiple neurodevelopmental disorders (including autism), psychiatric illnesses, and neurological disorders. These results may have implications for how changes in OFC selectivity drive motivational states and how a malfunctioning motivational system can drive neurodevelopmental and psychiatric illness due to chronic and enduring changes in these brain networks.

## Author Contributions

VT and IRO conceived of and designed the experiment. VT collected the data. VT performed statistical analysis for the task-based experiment and group-level resting state analyses. VT identified subject-specific face patch coordinates. CCD and AMM performed preprocessing and subject-level analysis of resting state experiment. VT and IRO interpreted the data and wrote the manuscript.

## Funding

This work was supported by a grant from the National Institute of Health to IRO (5R01MH073084) and NIH F32 (MH102024-01A1) to VT.

## Conflict of Interest Statement

The authors declare that the research was conducted in the absence of any commercial or financial relationships that could be construed as a potential conflict of interest.

## Footnotes

^1^www.fmrib.ox.ac.uk/fsl
